# Reactivation of the inactive X chromosome in development and reprogramming

**DOI:** 10.1007/s00018-012-1174-3

**Published:** 2012-09-30

**Authors:** Tatsuya Ohhata, Anton Wutz

**Affiliations:** 1 Wellcome Trust and MRC Stem Cell Institute, Department of Biochemistry, University of Cambridge, Tennis Court Road, Cambridge, CB2 1QR UK; 2 Present Address: Department of Molecular Biology, Hamamatsu University School of Medicine, 1-20-1 Handayama, Higashi-ku, Hamamatsu, 431-3192 Japan

**Keywords:** X chromosome inactivation, Dosage compensation, Mammalian development, Reprogramming

## Abstract

In mammals, one of the two X chromosomes of female cells is inactivated for dosage compensation between the sexes. X chromosome inactivation is initiated in early embryos by the noncoding *Xist* RNA. Subsequent chromatin modifications on the inactive X chromosome (Xi) lead to a remarkable stability of gene repression in somatic cell lineages. In mice, reactivation of genes on the Xi accompanies the establishment of pluripotent cells of the female blastocyst and the development of primordial germ cells. Xi reactivation also occurs when pluripotency is established during the reprogramming of somatic cells to induced pluripotent stem cells. The mechanism of Xi reactivation has attracted increasing interest for studying changes in epigenetic patterns and for improving methods of cell reprogramming. Here, we review recent advances in the understanding of Xi reactivation during development and reprogramming and illustrate potential clinical applications.

## Introduction

The inactive X chromosome (Xi) was originally observed as a dense staining structure in the nucleus of female cat neurons [1]. Since the original observation, the Barr body has inspired studies and served as a “visual” model for a silent chromatin state within the mammalian cell nucleus. Over the last 50 years, the process of X inactivation has kept its secrets. Although tremendous progress has been made by a number of laboratories, the mechanism behind X inactivation in its entirety remains to be worked out. The complexity of the overall mechanism, which also involves pathways that are known to be important for developmental gene regulation, has captured the attention of a growing number of scientists. The present text is placed in the background of a large number of recent review articles that have discussed the understanding of X inactivation in detail. We focus here on instances of reactivation of the Xi in development and cultured cells. This aspect appears to gain considerable importance as cell reprogramming requires the erasure of epigenetic patterns. Xi reactivation has been increasingly used as a model system for understanding chromatin changes during reprogramming and defining the reprogrammed pluripotent cell state. Reactivation of genes on the Xi has also been considered for ameliorating human genetic diseases caused by mutations on one of the two X chromosomes in female patients. Therefore, Xi reactivation is an emerging topic of interest beyond basic research with potential clinically relevant applications in the future.

In placental mammals, sex determination relies on the presence of the SRY gene on the Y chromosome for specifying male development. Few exceptions are known in which this sex determination system has evolved further by either Y chromosome loss (such as in certain species of spiny rats [2] and mole voles [3, 4]) or the evolution of dominant female determining X chromosomes (such as in wood lemmings [5]). As a consequence of male-limited transmission, meiotic recombination of the mammalian Y chromosome has been restricted and a large part of Y chromosomal sequences have eroded due to accumulation of mutations. Recent results analyzing the sex chromosomes of monotremes indicate that the mammalian XY system is relatively young with an estimated age of less than 166 million years [4, 6]. Notably, marsupial mammals share the X chromosome with placental mammals, whereas sequences of the monotreme sex chromosomes are unrelated. Importantly, the sequences of the mammalian X chromosome are identified on autosomes in monotremes, thus, providing compelling evidence that the mammalian XY system was derived from an ancestral autosome pair before the divergence of placental and marsupial mammals. An evolutionary origin closely before mammalian radiation has certain significance for explaining mechanistic differences in X chromosome inactivation (XCI) between mammalian species that have recently been appreciated. In both placental and marsupial mammals, the different number of X chromosomes between the sexes is compensated by inactivating one of the two X chromosomes in female cells. This leaves a single active X chromosome (Xa) in both males and females in the context of two of each autosomes [7]. Therefore, Y chromosome erosion and evolution of X inactivation must have led to a shift in gene dosage of X-linked genes relative to autosomal genes. As a consequence, selection pressure to maintain relative levels of gene products has led to increasing expression of X-linked genes. Recent evidence suggests that genes that contribute to multi-component complexes are most sensitive to changing X:A ratios [8]. The evolutionary progression of the mammalian XY system is likely complex, with selective forces acting in a heterogeneous manner on the many genes located on the X chromosome. In different mammalian species these selective forces, which can also include sexually antagonistic selection [9], have contributed to different patterns of gene inactivation, escape from XCI, gene loss, and translocations [10]. A significant amount of species differences has been uncovered in recent studies and has to be taken into account when extrapolating findings across mammals.

The mechanism of XCI has most extensively been studied in the mouse as an accessible model species for mammalian development. In mice, inactivation of the paternally inherited X chromosome is initiated at the 4-cell stage. This gives rise to an imprinted pattern of XCI in the extraembryonic (placental) tissues. At the late blastocyst stage, reactivation of the Xi is observed in cells that will form the epiblast. Therefore, two active X chromosomes (Xa) are observed in the cells of the developing embryo between embryonic day 4.5 (E4.5) and E5.5. At the onset of gastrulation, inactivation of either the paternal or the maternal X chromosome establishes dosage compensation in embryonic tissues. Once random X inactivation is initiated, all progeny of the cell maintain the same pattern of either a maternal or paternal Xi [7]. This leads to a genetic mosaic of cells with opposite XCI patterns. It has been estimated that the embryo contains approximately 200 cells when this genetic mosaic is established [11]. The presence of cells with expression from either X chromosome makes female mammals robust against deleterious mutations on one of the X chromosomes [12]. This compensation is not possible in males and a number of X-linked mutations either lead to lethality in males or can cause diseases [12]. Random XCI has therefore a positive effect on female fitness in placental mammals, which might be significant, as maternal fitness is of critical importance in placental systems [13, 14]. Marsupial mammals possess exclusively imprinted XCI of the paternal inherited X chromosome and thus do not benefit from increased female fitness through XCI mosaicism. It has been argued that imprinted XCI could be an ancestral form of XCI with the advent of random XCI as a result of reproductive selective pressure. However, there is scant evidence in support of this hypothesis and a parallel evolution of both mechanisms should be considered equally likely.

## Initiation of chromosome-wide gene silencing

**Fig. 1:**
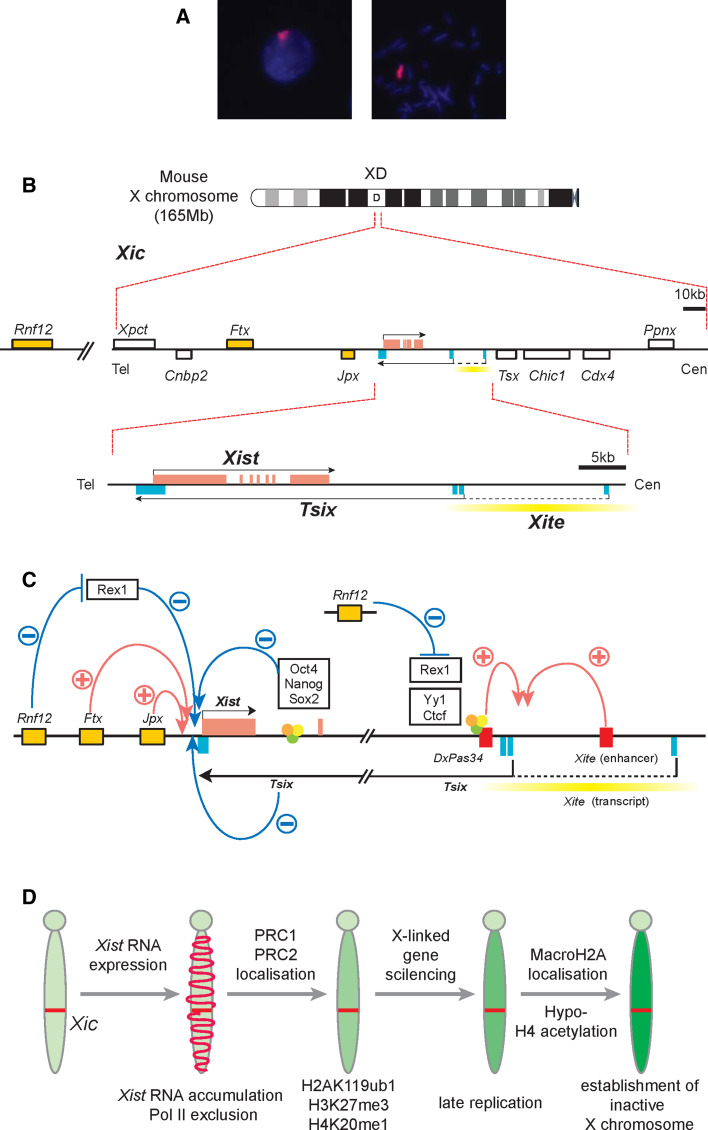
The genes involved in regulation of X inactivation. **a** Mouse *Xist* RNA in interphase (*left*) and metaphase (*right*). *Xist* RNA (*red*) initiates XCI in *cis*. **b** The mouse X inactivation center (*Xic*) contains the *Xist* gene and regulatory elements. **c** The non-coding RNAs *Ftx* and *Jpx* positively regulate *Xist* expression, whereas *Tsix* is a repressor of *Xist* and is transcribed in antisense orientation over the *Xist* locus. *Rnf12* is also an activator of *Xist* and might exert its function through targeting the transcription factor Rex1 for degradation. Rex1 represses *Xist* expression via *Tsix*-dependent and -independent mechanisms. The transcription factors Oct4, Nanog and Sox2 bind to a site within *Xist* intron 1, and are thought to repress *Xist*. *Tsix* expression is also regulated by factors in pluripotent cells including Rex1, YY1 and Ctcf. *Xite* has been identified as an enhancer of *Xist* that also produces non-coding transcripts. **d** XCI is a complex process that involves a series of sequential steps

In placental mammals, XCI requires the *Xist* gene (Fig. 1a). *Xist* is located on the X chromosome and is specifically expressed from the Xi. Its product is a noncoding RNA that accumulates within the chromosome territory of the Xi. *Xist* is required for chromatin modifications and gene repression on the Xi. Notably, *Xist* is specific for placental mammals with no orthologous RNA described in marsupial mammals or any vertebrate species to date [15–17]. Recently, the noncoding RNA gene *Rsx* (*RNA*-*on*-*the*-*silent X*) has been identified in marsupials. *Rsx* shares some properties with *Xist*, even though its sequence is unrelated to *Xist* [18]. Similar to *Xist* in placental mammals, *Rsx* localizes to the marsupial Xi. It has further been shown that *Rsx* expression can cause gene repression when expressed in mouse cells. This suggests that *Rsx* has evolved independently from *Xist* for the marsupial dosage compensation system and this case of convergent evolution might provide opportunities for studying the function of non-coding RNAs in chromatin regulation. In mice, *Xist* expression is regulated by genetic loci within its surrounding chromosomal locus that is referred to as the *X inactivation center* (*Xic*, Fig. 1b). Sequences within the *Xic* provide signals that allow to establish the number of X chromosomes per cell. It has been shown that *Xic* sequences when transgenically transferred to autosomes can induce X chromosome inactivation in male mouse ES cells [19, 20]. Deletion of sequences within the *Xic* has shown to lead to differential effects. Deletion of *Xist* sequences [21–23] results in abrogation of XCI on the deletion bearing the X chromosome and in inactivation of the alternative X chromosome. In contrast, deletions in the 3′-region of the *Xist* gene are associated with a preferential activation of *Xist* and inactivation of the deletion-bearing chromosome [24]. Within this 3′ region lies the promoter and regulatory elements for expression of the *Tsix* noncoding RNA (Fig. 1b). *Tsix* is transcribed in antisense orientation of *Xist* [25] and acts as a repressor of *Xist* expression [26]. Forced expression of *Tsix* blocks *Xist* expression and XCI [27]. Other noncoding RNAs have also been implicated in XCI regulation. These include *Xite*-derived RNAs [28], *Jpx/Enox* [29], and *Ftx* [30]. *Xist* expression is also regulated by transcription factors (Fig. 1c). Binding sites for OCT4, SOX2, and NANOG within *Xist* intron 1 have been implicated in repression of *Xist* in mouse ES cells [31]. Further binding sites around the promoter of *Tsix* are implicated in modulating *Tsix* expression and thereby influencing a repressive effect on *Xist* [31, 32]. The RNF12 protein is an activator of XCI and is expressed from a locus with close linkage to *Xist*. This is the first candidate protein that has features of a counting factor for X chromosomes. RNF12 levels correspond to the probability that a given cell initiates XCI [33–35]. A recent study links RNF12 to *Rex1* [36], a transcription factor that has been implicated in the expression of *Tsix* and *Xist* regulation, thus, providing a potential mechanism (Fig. 1c; [37]). In addition, inter-chromosomal pairing of *Xic* loci has been implicated in the process of counting the number of X chromosomes. Two pairing elements have been described. The *Tsix* promoter region acts as a pairing element when introduced transgenically into mouse ES cells [38, 39]. A second pairing region is located around the *Xpr* region upstream of *Xist* and is reported to support an independent and possibly earlier pairing event [40]. The *Xpr* region has been shown to be capable of inducing trans-chromosomal pairing. However, its relevance as an activator for XCI is debated [33, 41]. It appears that multiple regulatory input converges on the *Xist* promoter to ensure that all but one X chromosomes are inactivated per diploid genome. Conversely, *Xist* expression is prevented from the future Xa.

Once *Xist* expression is activated, the RNA accumulates over the Xi chromosome territory and mediates chromatin modifications (Fig. 1d). Depletion of factors associated with transcription from the Xi territory are the first changes that can be detected [42, 43]. These include the loss of RNA polymerase II and nascent transcripts. Loss of activating histone marks such as histone H3 lysin 4 tri-methylation (H3K4me3) and acetylation of histone H3 (H3ac) are followed by a gain in chromatin marks associated with Polycomb group (PcG) complex activity (reviewed in [44]). Polycomb repressive complex 1 (PRC1) mediates mono-ubiquitination of histone H2A lysine 119 (ubH2A) and PRC2 catalyses tri-methylation of histone H3 lysine 27 (H3K27me3) on the Xi. Notably, these changes in chromatin modifications are not sufficient to cause gene silencing. It has been shown that chromatin modifications but not gene repression is effected by expressing a mutant *Xist* RNA that lacks sequences on the 5′-end [42, 45–47]. The *Xist* 5′ region contains a sequence motif that is conserved among all placental mammals and has been termed repeat A element. Seven to eight repeats of a core motif are separated by a variable spacer [47]. It is presently thought that *Xist* repeat A triggers additional pathways for gene silencing [48], but association with Polycomb group complexes and structural aspects are also discussed [49–51]. In the presence of repeat A, *Xist* represses genes and silent genes associate with the modified chromatin in the center of the Xi chromosome territory [42]. Thus, a reorganization of genic chromatin correlates with gene silencing in XCI. If chromatin reorganization is causal for repression or a consequence is presently unclear [42, 52, 53].

The Xi chromatin is further modified when cellular differentiation progresses (Fig. 1d). This involves the recruitment of additional factors. It has been shown that the histone variant macroH2A [54–57], the Trithorax group protein Ash2L [46], and the scaffold factor SAF-A [46, 58, 59] become enriched on the Xi in differentiated cells. In addition, DNA methylation of Xi-linked promoters is established in a manner that depends on SmcHD1 and Dnmt1 [60, 61]. A number of epigenetic mechanisms act together to endow gene repression on the Xi with a remarkable stability. Reactivation of genes on the Xi has been shown to require the combined interference with DNA methylation, histone deacetylases (HDACs), and *Xist* [23]. Reactivation of genes on the Xi in somatic cells is heterogeneous and no case of reactivation of the entire chromosome has been documented. Importantly, *Xist* is not critical for gene repression in mouse or human somatic cells [62, 63]. Thus far, chromosome-wide Xi reactivation has only been achieved by reprogramming of somatic cells to pluripotent stem cells [64].

## X inactivation and Xi reactivation in development

**Fig. 2:**
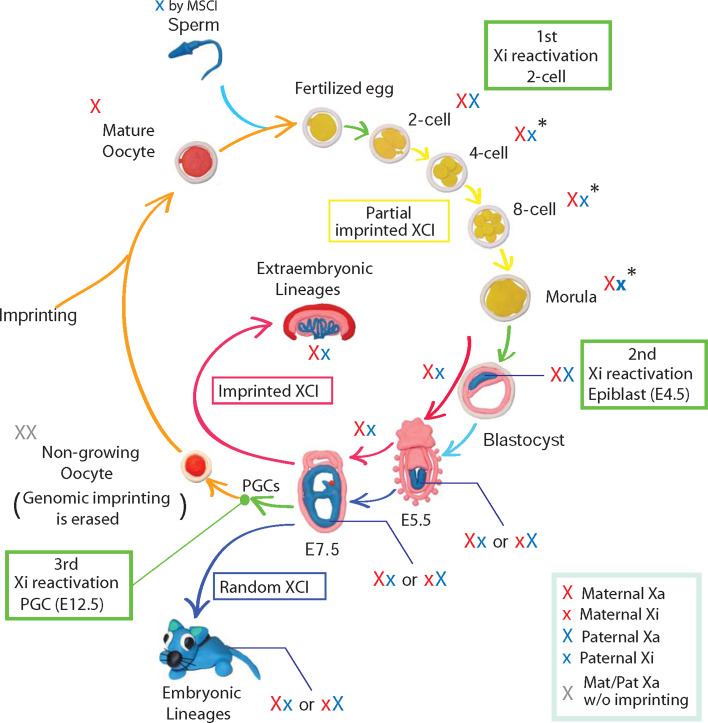
Dynamic activity of the X chromosome in mouse development. The unpaired X chromosome is silenced during spermatogenesis by MSCI. After fertilization reactivation of X-linked genes is observed at 2-cell stage from the paternal X chromosome (1st wave of Xi reactivation, *green arrow*). During female preimplantation development, the paternal X chromosome becomes inactivated (*yellow arrows*), whereby gradual progression of XCI is completed in the developing extraembryonic lineages (imprinted X-inactivation, *red arrows*). In contrast, the paternal Xi is reactivated in cells of the developing epiblast in the late blastocyst (2nd wave of Xi reactivation, *green arrow*). Subsequently, the maternal or the paternal X chromosome is randomly chosen to be inactivated during embryogenesis (random X-inactivation, *blue arrows*). In primordial germ cells (PGCs), Xi is reactivated is associated with reprogramming of epigenetic patterns including genomic imprinting (3rd wave of Xi reactivation, *green arrow*). Xi reactivation occurs before oogenesis is initiated. During the maturation of oocytes new genomic imprints are established for marking maternal alleles in the next generation (*orange arrows*). Therefore, the passage of the mouse X chromosome through a generation can involve multiple changes between active and inactive states highlighting periods of epigenetic reprogramming

A number of studies have provided detailed insight into the initiation of XCI in mouse embryogenesis (Fig. 2). In mice, the paternal X chromosome (Xp) is always chosen as Xi giving rise to imprinted XCI in preimplantation development and the extraembryonic lineages. To explain the difference between the two parental X chromosomes, it has been proposed that opposing epigenetic marks are established in the parental germ lines within the *Xic*. Embryos with X chromosome aneuploidy have been used to investigate the nature of these imprinting marks and to establish whether they originate from oocyte, sperm, or both. Embryos that inherit a paternal X chromosome (Xp) but lack a maternal X chromosome (Xm) due to missegregation in oocyte development can survive, indicating that inactivation of Xp can be prohibited if no Xm is present. Therefore, the imprint that forces Xp to inactivation is at least reversible or alternatively is absent altogether [21, 65]. In contrast, if two maternal X chromosomes (Xm) are inherited from oocytes, XCI is not initiated, resulting in embryos with two active Xm [66]. This suggests that the Xm is marked for preventing inactivation and that this marking is not reversed in early mouse development. The establishment of an imprint on Xm during oogenesis was further confirmed by nuclear transfer experiments [67]. The molecular nature of the imprint on Xm remains to be defined. Notably, and in contrast to many other imprinted gene clusters such as *Igf2r*, *Kcnq1*, *Pws/As*, *Gnas*, *Igf2*, *Dlk1*, and *Grb10* [68], DNA methylation is not required for imprinted *Xist* expression [69, 70].

During spermatogenesis, unsynapsed X and Y chromosomes are silenced through meiotic sex chromosome inactivation (MSCI) [71] and it has been suggested that the Xp arrives in a partially silenced state at fertilization. The absence of nascent transcripts from the Xp territory in 2-cell embryos has been used as an argument for a carry-over effect of MSCI-mediated Xp silencing through fertilization [72]. These experiments were performed using repeat-derived RNA in situ hybridization probes that can identify domains of transcription within both genic and intergenic regions. Since repetitive elements are largely absent from coding regions, they preferentially detect intronic or noncoding transcription but not necessarily expressed genes. Although some carryover of MSCI can be detected, it has been shown that MSCI-mediated inactivation is not required for inactivation in preimplantation embryos. Okamoto et al. [73] used a YAC transgene, which contains sequences of the *Xic* and was integrated into an autosome. *Xist* expression of these YAC transgenes was observed from the paternal genome in extraembryonic lineages to cause inactivation of the autosomal chromatin. In this case, MSCI can be ruled out as the transgenic autosome is normally paired in male meiosis. Further studies also observed that genes on the Xp become temporary reactivated in 2-cell embryos indicating that the carryover of MSCI-mediated Xp silencing is only temporary and might have little effect on genes [73, 74]. A recent report by Namekawa et al. [75] finds that Xp-linked genes are temporarily reactivated and subsequently inactivated in preimplantation development. Taken together, these studies demonstrate that genes on the Xp are activated in 2-cell stage simultaneous with the timing of a wave of zygotic genome activation (EGA) [73, 75]. The mechanism for the Xp reactivation after fertilization is unclear. However, it may be linked with the process of repackaging of sperm DNA into chromatin. In this regard, the recent observation of Tet3-dependent conversion of 5-methyl cytosine (5mC) to 5-hydroxymethyl cytosine (5hmC) on the paternal genome in zygotes might also be of significance [76]. The differential marking of both parental genomes in the mouse preimplantation embryo can be expected to influence the expression of imprinted genes, including *Xist*.

Genes on Xp are subsequently gradually silenced once *Xist* expression is activated. *Xist* has been observed to become activated at the 2-cell stage leading to the first signs of Xp gene silencing around the 4-cell stage. Thereby, gene silencing appears to be pronounced over positions close to the *Xic* [72, 74, 75]. It has been shown that a deletion of the *Xist* gene in mice leads to embryonic lethality after implantation [21, 22]. However, the requirement of *Xist* for imprinted XCI before preimplantation of the embryo has been subject to some controversy. A study by Katalanty et al. [77] observed silencing of genes on an Xp carrying a deletion of the *Xist* gene. Furthermore, Williams et al. showed that *Xist* depletion in *Grb2*−/− blastocysts, which is associated with a wider expression pattern of *Nanog*, does not increase the number of blastomeres, which showed biallelic expression of X-linked genes, suggesting that imprinted XCI observed in blastocysts is independently regulated from *Xist* accumulation [78]. However, Namekawa et al. [75] observe that Xp inactivation is controlled by *Xist*. At present, the discrepancy between these results cannot be easily reconciled as it appears that the same experimental setup and techniques resulted in conflicting observations [75, 77]. One interpretation of these findings may be that silencing of some but not all genes requires *Xist* [75].

In addition to studies in mice, XCI has also been investigated in other mammalian species including rabbit and human preimplantation embryos. This has led to surprising observations of species-specific differences. *Xist* expression from both Xm and Xp is observed in rabbit blastocysts [79], indicating that rabbits might not have imprinted XCI. In this case, XCI is initiated simultaneously on both X chromosomes followed by reactivation of one X chromosome in a random manner. Notably, *XIST* expression from both X chromosomes was also observed in a study of human blastocysts [79]. *XIST* expression did not initiate gene repression in human blastocysts and, thus, XCI appeared to be established only after implantation. However, these results may need independent confirmation, as earlier work in human preimplantation embryos reported that XCI is initiated before the blastocyst is formed in a manner involving upregulation of *XIST* from one X chromosome [80]. These discrepancies may reflect difficulties in obtaining and culturing human preimplantation embryos. To resolve this issue, an assessment of XCI in non-human primates might be of high interest. However, reprogramming of the X chromosomes to an active state after fertilization similar to autosomes appears a conserved feature among placental mammals.

### Requirement for dosage compensation in the mouse embryo

In mice, deletion of *Xist* on the Xp leads to developmental arrest of female embryos but does not appear to disrupt preimplantation development. Embryos carrying a paternally inherited mutation of *Xist* develop into cytologically normal blastocysts that can implant [21]. It has been argued that *Xist* might not be required for dosage compensation before implantation [78]. More recent evidence indicates that indeed lack of dosage compensation can be tolerated for most expressed X-linked genes prior to implantation [75]. In addition, parthenogenetic blastocysts and haploid mouse embryos have been used for the establishment of stem cell lines, confirming that lack of dosage compensation can be tolerated to a large extent in early embryonic cell types [81, 82]. In the case of haploid embryos, a relative ratio of X-linked to autosomal (X:A) gene expression of 1 to 2 cannot be achieved and therefore these embryos reflect a non-dosage compensated state.

In contrast, post-implantation development depends critically on proper dosage compensation. Embryos carrying a deletion of *Xist* on the Xp arrest at E7.5 soon after implantation [21]. This result indicates that inactivation of the paternally inherited X chromosome is critical for postimplantation development and cannot be compensated by inactivation of Xm. As a consequence, two X chromosomes are active in the extraembryonic lineages and lack of dosage compensation leads to defects that impair embryo development. The extraembryonic lineages include the visceral and parietal endoderm, which are derived from the primitive endoderm, and parts of the placenta including trophoblast giant cells, spongiotrophoblast, and syncytiotrophoblast that are derived from the trophectoderm [83, 84]. Primitive endoderm is thought to be derived from the hypoblast that is formed from cells of the inner cell mass (ICM) before reactivation of the paternal Xi is accomplished in the blastocyst. Interestingly, parietal endodermal cells have been established by overexpression of Gata6 gene in mouse ES cells, which showed random X inactivation but not imprinted X inactivation [85]. This result is consistent with the notion that imprinted XCI in the primitive endoderm lineage is maintained from preimplantation embryos and reactivation might not occur during normal development.

Repression of *Xist* on the maternal X chromosome by *Tsix* is required for imprinted XCI and development. Female embryos carrying a mutation of the *Tsix* gene on Xm show embryonic lethality owing to ectopic *Xist* expression and inactivation of Xm as well as Xp [86]. The Rnf12/RLIM E3 ubiquitin ligase has been identified as a dosage-dependent *Xist* activator [33]. *Rnf12* is located on the X chromosome and appears to regulate imprinted XCI [35]. It has been observed that the maternal but not paternal transmission of a mutation in *Rnf12* prevents *Xist* activation and results in embryonic lethality [35]. Thus, a mutation of *Rnf12* on Xm and a mutation of *Xist* on Xp result in defects in imprinted XCI and similar phenotypes in female embryos [21, 35]. Furthermore, particularly high levels of RNF12 protein were observed in pronuclei of zygotes [35], suggesting that maternally deposited RNF12 may contribute to the initiation of imprinted XCI. Notably, the transmission of the wild-type X chromosome, but not *Rnf12* mutant X chromosome, from *Rnf12+/−* oocytes can give rise to normal female offspring at the expected Mendelian ratio [35]. Therefore, expression of *Rnf12* from maternal X chromosome throughout ovulation and after fertilization (zygotic activation) are essential for the activation of *Xist* expression from the paternal X chromosome.

### Imprinted XCI in mouse development

Imprinted XCI is maintained in the extraembryonic lineages that give rise to the extraembryonic membranes and contribute to the placenta. Inactivation of genes on the Xp in these lineages is heterogenous. It has been found that an *Atrx* mutation on Xm, a member of the SNF2 family of ATPase/helicase proteins, leads to escape of the paternally inherited *Atrx* gene from imprinted XCI [87]. Escape from imprinted XCI is not common among other X-linked genes such as *Dkc1*, *G6PD*, and *Chm* [88–90], indicating that silencing of the majority of genes on Xp is strictly maintained. However, XCI in the extraembryonic lineages might be less stable than in embryonic lineages. Spontaneous reactivation of a GFP transgene has been reported in a subset of trophoblast giant cells [91] and parietal endoderm [27], whereas no reactivation was observed in embryonic lineages [63]. In contrast to embryonic lineages, maintenance of Xi silencing requires *Xist* expression in trophoblast and parietal endoderm [27]. Furthermore, the Polycomb group protein *Eed* is required for maintaining *Xist* expression in trophoblast stem (TS) cells [92]. It has been shown that differentiation of *Eed*-deficient trophoblast stem cells is accompanied by reactivation of genes on the Xi. However, *Eed* is not essential for maintaining XCI in the embryonic lineages suggesting different molecular requirements between embryonic and extraembryonic cells [93]. On the contrary, DNA methylation appears to be essential for maintenance of XCI specifically in the embryo. Whereas a critical role for *Dnmt1* and *SmcHD1* in maintenance of Xi silencing in the embryo has been established, DNA methylation appears largely dispensable for imprinted XCI [60, 61]. These observations indicate that maintenance of XCI depends on different epigenetic pathways in different cell lineages. Long-term maintenance of dosage compensation might be less critical in placental lineages as these are dispensable after birth and the view emerges that the chromatin configuration of the paternal Xi in early mouse embryos might be conducive to reactivation.

### Xi reactivation in mouse epiblast development

The switch from imprinted to random XCI in the mouse embryo requires the reactivation of genes on the Xp [72, 75, 94]. Reactivation occurs in inner cell mass (ICM) cells of the blastocyst at E4.5 that give rise to the developing epiblast lineage. Xp reactivation is accompanied by loss of *Xist* expression between the early and late blastocyst stage [95] followed by loss of H3K27me3, EZH2, and EED from the Xi [43]. Notably, Williams et al. [78] observed reactivation of genes on the Xp shortly before *Xist* RNA and H3K27me3 were lost from the paternal Xi, suggesting that gene reactivation occurs when *Xist* and H3K27me3 are present on the Xi. This observation is at first sight surprising, as an earlier study has shown that *Xist* is able to initiate gene repression in ES cells that are derived from ICM cells [47]. A likely explanation could be that ES cells do not resemble all aspects of the developing ICM and, thus, in vivo and in vitro results could be different. Alternatively, loss of *Xist* and H3K27me3 from the chromosome might be delayed, leading to the detection of residual signals in few cells in the embryo.

*Tsix* has been implicated in the switch from imprinted to random XCI through inducing efficient H3K4 methylation over the entire *Xist*/*Tsix* unit for equalizing and resetting the epigenetic status on both *Xic* alleles [96]. It has been shown that transient induction of *Tsix* expression from Xp in blastocysts represses *Xist* and leads to Xp reactivation [27]. However, *Tsix* induction does not induce a switch from imprinted to random XCI in the extraembryonic lineages and when *Tsix* expression is terminated inactivation of the same X chromosome is restored. This result indicates that additional steps are required for resetting the imprint on Xm for switching to random XCI.

The transcription factors OCT4 (official gene nomenclature *Pou5f1*, also called *Oct3/4*), SOX2, and NANOG have been proposed to repress *Xist* when pluripotency is established [31]. These three factors bind to a site within *Xist* intron 1. Consistent with a role in *Xist* repression, it has been shown that the loss of OCT4 or NANOG leads to activation of *Xist* expression in mouse male ES cells. However, a genetic deletion of the binding site within *Xist* intron 1 does not result in a similarly strong effect on *Xist* expression suggesting that other regulators might also be involved [34]. In line with this finding, a combined deletion of the intron 1 binding site and *Tsix* has been observed to enhance *Xist* upregulation suggesting a synergistic effect [97]. Even though intron 1 element is not absolutely required for repressing *Xist* in ES cells, it may still function in switching imprinted to random XCI in the embryo. Future work will be crucial to investigate whether blastocysts carrying a paternal deletion of the intron 1 element have defects in resetting imprinted XCI. Recent work has shown that RNF12 regulates the protein level of REX1 that has been implicated as a transcription factor for the activation of *Tsix* [36, 37]. These findings might provide an additional link between XCI and the transcription network of pluripotent cells. Notably, *Rex1* expression is rapidly lost when ES cells enter differentiation and, thus, correlates negatively with XCI, suggesting that reduced activation of *Tsix* through loss of REX1 might contribute to the initiation of XCI.

In conclusion, reactivation of the Xi in the ICM appears to be tightly linked to the establishment of pluripotency. Imprinted XCI appears not to be stable at this stage, and might be readily reversible following loss of *Xist* expression and chromatin modifications [27, 98]. However, ES cells can also reactivate a somatic Xi, indicating that the chromatin environment of pluripotent cells facilitates changes of epigenetic states. This observation is in line with reports of a more dynamic chromatin structure and particular chromatin modifications associated with active chromatin in mouse ES cells [99, 100]. Reactivation of imprinted XCI might be mediated by a combination of a reversible chromatin structure on the paternal Xi and active mechanisms that remodel silent chromatin and repress *Xist*.

### Establishment of XCI in the developing mouse embryo

Following the reactivation of paternal X chromosome in epiblast lineages, either the maternal or paternal X chromosome is randomly chosen to be inactivated. A number of studies have contributed to an understanding of the mechanism of choosing the Xi (reviewed in [44]). *Xist* expression from maternal X chromosome is first observed at E5.5, suggesting the initiation of random XCI [94]. Formation of Xi heterochromatin has been investigated in the mouse embryo using different cytological methods. Rastan et al. [101] showed that an Xi could be observed in E7.0 epiblast using Kanda’s method, which visualizes Xi as a dark-stained chromosome. Similarly, Takagi et al. [102] concluded that an Xi could already be observed in E6.5 embryos taking advantage of late replication as a marker of the Xi. Consistent with this timing of XCI, a limited number of X-linked genes, including *Hprt* and *Pgk1* as measured by their enzymatic activity, are also silenced on the Xi in E6.5 and E7.0 embryos, respectively [11, 103]. In addition, DNA methylation of the *Pgk1* promoter on the Xi is observed around E7.0 [104]. Silencing of *Pgk1* and establishment of DNA methylation on the *Pgk1* promoter occur simultaneously, whereas DNA methylation of the *Hprt* promoter is first observed at E13.5, which is a considerable amount of time after *Hprt* silencing is observed at E6.5 [105]. This finding suggests that initially *Hprt* repression is largely independent of DNA methylation before E13.5. Taken together, these studies indicate that dosage compensation is established by E6.5 in the female mouse embryo.

Several factors have been investigated for a potential role in the maintenance of gene repression on the Xi in later embryos (reviewed in [44]). The SmcHD1 (structural maintenance of chromosomes hinge domain containing 1) protein localizes to the Xi and is required for the maintenance of gene silencing on the Xi [60]. It has been shown that genes on the Xi become activated in *SmcHD1*-deficient embryos. In addition, DNA methylation is lost on promoters of some genes on the Xi. This is further consistent with a requirement for the DNA methyltransferases Dnmt1 for maintaining gene silencing on the Xi [61]. Although Polycomb group (PcG) complexes are recruited to the Xi, their role in maintaining silencing remains to be clearly established. Recruitment of PcG proteins appears to be largely dependent on *Xist* [106, 107], which is required for initiation but not for maintenance of the Xi in differentiated cells [62, 98]. These observations indicate that most PcG proteins that have been studied thus far at the Xi might actually not be essential for maintaining random XCI. However, it is conceivable that other components or specific chromatin structures are established through PcG complexes that are maintained in the absence of *Xist* and, thus, could facilitate maintenance of XCI. Xi reactivation is observed when a genetic disruption of *Xist* is combined with chemical inhibition of DNA methylation and histone deacetylation with 5-aza deoxycytosine (5-azadC) and Trichostatin A (TSA), respectively [23]. This observation suggests that multiple epigenetic mechanisms contribute to gene repression on the Xi in somatic cells. Reported attempts for gene reactivation by interference with these mechanisms generally lead to reactivation of a small number genes in a small percentage of cells, indicating that loss of repression on the Xi can be induced in a stochastic but not a chromosome-wide manner. Chromosome-wide Xi reactivation has to date been achieved through reprogramming of somatic cells to induced pluripotent stem cells (iPS cells).

### Xi reactivation in primordial germ cell development

In mice, primordial germ cells (PGC) can be first identified at E7.5 in a posterior position of the epiblast. PGCs originate from the epiblast that has already initiated random XCI and migrate to the genital ridge by E10.5 (reviewed in [108]). During this migration, epigenetic modifications are reprogrammed and the Xi is reactivated before sex-specific differentiation of the germline is initiated at E12.5. A series of experiments have defined the timing of Xi reactivation in PGC development using single-cell PCR- and PGC-specific markers [109–111]. These investigations have led to the conclusion that Xi reactivation is initiated during the migration of PGCs towards the genital ridge. Thereby, reactivation proceeds in a gradual manner over a considerable developmental interval. The effect of Xi reactivation is that both X chromosomes in female PGCs are in an active state before oogenesis is initiated.

The mechanisms of Xi reactivation in PGC development has attracted considerable interest and has been investigated by a number of studies. Logically, it can be divided into several steps: repression of *Xist* expression, loss of Xi marker H3K27me3 accumulation, and reactivation of X-linked genes from Xi. Reactivation of genes on the Xi appears to be a gradual process that also temporally overlaps with phases of DNA demethylation and changes in chromatin modifications that are associated with genome-wide reprogramming of PGCs [108]. Interestingly, the timing of the initiation of *Xist* repression and *Nanog* expression appears to overlap in E7.5 PGCs [112] similar to the ICM, when *Nanog* is required for the establishment of pluripotency [113]. Repression of *Xist* expression from imprinted paternal Xi is observed in NANOG-positive cells of the ICM [94]. Furthermore, NANOG expression is correlated with Xi reactivation and *Xist* repression at the transition from pre iPS cells to iPS cells [114]. Taken together, *Nanog* expression may be a candidate for factors contributing to *Xist* repression in PGCs, similar to the role of pluripotency factors for *Xist* repression in the ICM has been proposed [31]. Loss of H3K27me3 from the Xi may be explained as a consequence of loss of *Xist* expression, since it has been shown that PcG recruitment depends on *Xist* in ES cells [45, 106]. However, it remains to be elucidated whether *Xist* repression is actually required for Xi reactivation in PGCs. A number of epigenetic modifications, including H3K9me2, H3K27me3, H2A/H4R3me2s, and DNA demethylation, undergo prominent changes during PGC development and are thought to mediate reprogramming of the germline genome [108]. In addition, DNA demethylation is observed. This makes PGC development an interesting system for analyzing epigenetic processes and the mechanistic understanding of Xi reactivation.

## Exploring the mechanism of Xi reactivation

In mice, three distinct developmental stages are associated with Xi reactivation. The paternal X chromosome is reactivated in the oocyte after fertilization and thereby MSCI is reversed. Following imprinted XCI in preimplantation development, Xi reactivation is observed in the developing epiblast at E4.5. Finally, in migrating PGCs, Xi reactivation establishes two active X chromosomes in female germ cells before oogenesis. During this process, genomic imprinting is erased, whereas imprints are maintained through fertilization and epiblast development (Table 1).

**Table 1:** Functional differences between X reactivation in vivo

	Status	Imprinted XCI	Genomic imprinting
X reactivation in oocyte	Totipotency	Maintained	Maintained
X reactivation in epiblast	Pluripotency	Erased	Maintained
X reactivation in PGC	Unipotency	–	Erased

**Fig. 3:**
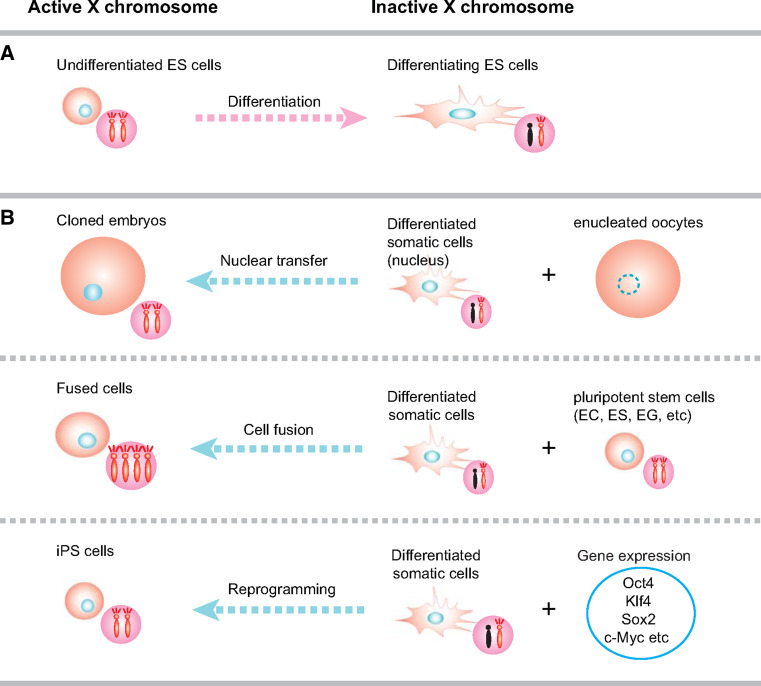
Cellular systems recapitulating Xi reactivation. **a** Mouse female ES cells possess two active X chromosomes. Upon differentiation, random XCI is initiated. **b** Reactivation of the Xi of mouse somatic cells can be achieved by a number of experimental manipulations. Xi reactivation is recapitulated during somatic cell nuclear transfer into oocytes, cell fusion with pluripotent cells such as mouse ES cells and EG cells, and iPS cell reprogramming

The mechanisms of Xi reactivation during these cell reprogramming episodes remain to be elucidated. It will be important to establish if common pathways can be identified or entirely different pathways are used at specific developmental stages. For addressing reprogramming mechanisms, different experimental systems have been developed (Table 2). Xi reactivation is recapitulated during nuclear cloning [115], cell fusion with pluripotent cells [116], and iPS cell generation through the expression of pluripotency factors (Fig. 3; Table 2; [117]). In addition, genetic and chemical screens have been applied for identification of factors that lead to reactivation of genes on the Xi.

**Table 2:** X reactivation observed by in vitro manipulation

Source	Method	Confirmation	Assumed mechanism in vivo	Species	Years	References	Note
Thymocyte, bone marrow cells	Cell fusion with EC cells (cell fusion)	Replication timing, X-linked gene reactivation (Pgk-1)	Reactivation in epiblast?	Mouse	1983	[83]	
Thymocyte	Cell fusion with EG cells (cell fusion)	Replication timing	Reactivation in PGC cells?	Mouse	1997	[131]	Genomic imprinting is erased
Tail tip fibloblast, cumulus cells	Nuclear transfer to oocyte (nuclear transfer)	X-linked gene reactivation (X-linked GFP transgene)	Reactivation in Oocyte?	Mouse	2000	[115]	
Thymocyte	Cell fusion with ES cells (cell fusion)	Replication timing, unstable *Xist* transcription	Reactivation in epiblast?	Mouse	2001	[129]	Genomic imprinting is maintained
Embryonic fibloblast, tail tip fibloblast	Gene expression, Oct4, Sox2, c-Myc, Klf4 (generating iPS cells)	*Tsix* and *Xite* expression, *Xist* repression, loss of histone marks, X-linked gene reactivation (Pgk-1)	Reactivation in epiblast?	Mouse	2007	[117]	Switch to random XCI
Neural stem cells	Gene expression, Oct4, Klf4 and 2i medium (generating iPS cells)	Loss of H3K27me3 accumulation	Reactivation in epiblast?	Mouse	2008	[114]	
Fibroblast, ESCs	Gene expression, Oct4, Sox2, Klf4, and Klf2 and 2i medium with LIF (generating iPS cells)	*Xist* expression (lack of *Xist* clouds by FISH), methylation status on the *Xist* promoter (methylated)	Reactivation in epiblast?	Human	2010	[138]	
EpiSCs	Nuclear transfer to* Xenopus* oocyte (nuclear transfer)	*Xist* repression, X-linked gene reactivation (X-linked GFP transgene)	Reactivation in Oocyte?	Mouse/*Xenopus*	2011	[125]	
Neonatal and adult fibloblast	Gene expression, 6 factors (Oct4, Sox2, Klf4, c-Myc, RAR-γ, Lrh-1) and 2i medium with LIF (generating iPS cells)	*Xist* repression and increasing X-linked genes (CXORF15, PLS3, RBBP7, UTX) by qRT-PCR	Reactivation in epiblast?	Human	2011	[139]	
EpiSCs	Gene expression, Prdm14 and Klf2	*Tsix* expression, *Xist* repression, X-linked gene reactivation (X-linked GFP transgene)	Reactivation in PGC cells or epiblast?	Mouse	2012	[142]	Genomic imprinting is maintained

### Triggering Xi reactivation by nuclear transplantation into oocytes

Transplantation of cell nuclei into oocytes of frogs and mammals has been used to reset the developmental potential of embryonic and somatic cells for producing cloned animals [118–120]. A somatic nucleus transferred into enucleated oocytes can recapitulate the reprogramming process observed in a fertilized egg. Analysis of XCI in cloned mice has shown that if a somatic donor cell is used, the donor cell’s Xi is chosen for inactivation in extraembryonic tissues of cloned embryos similar to imprinted inactivation of the paternal X chromosome in embryos obtained from fertilization [115]. In the embryonic cells of cloned mice random XCI is observed suggesting normal reactivation followed by random XCI in the epiblast can be accomplished. Notably, if female ES cells that possess two active X chromosomes are used as nuclear donor, random XCI is also observed in the extraembryonic tissues of cloned mice [115]. This observation indicates that a counting mechanism for random XCI or stochastic choice can substitute in the extraembryonic lineages for an imprint or pre-determined inactivation state. This is further consistent with the development of parthenogenetic and androgenetic female embryos that possess exclusively either maternal or paternal X chromosomes. Furthermore, the kinetics of Xi reactivation in cloned embryos, as observed using an X-linked GFP transgene, is consistent with physiological Xi reactivation in normal embryogenesis [115]. Changes in histone modifications [121] and DNA demethylation [122] have been studied in nuclear transfer embryos. These studies suggest that subtle differences exist possibly owing to technical limitations or different donor cell states. However, the observations suggest that cloning recapitulates reprogramming of the zygotic genome in fertilized egg development to a large extent. Recently, the role of the Tet3 enzyme for hydroxymethylation of DNA of the paternal genome has been demonstrated [76]. This might be one of several mechanisms that mediate reprogramming of the genome after fertilization. A major difference between the genomes in sperm and in somatic cells is their packaging into chromatin. The sperm genome is re-packaged into chromatin after fertilization and might be more accessible for modifications in the zygote than a genome transferred into the oocyte from somatic cells.

Nuclear transfer in mammals is an inefficient process owing to erroneous reprogramming of the donor genome. Incomplete erasure of epigenetic information from somatic cells, genetic aberrations and technical problems with embryo viability are key factors to consider. In addition, down-regulation of X-linked genes is observed in both male and female cloned mouse embryos. This has been linked to an inappropriate activation of *Xist* and the initiation of XCI. Notably, the efficiency of mouse cloning is significantly improved when nuclear donor cells bearing a deletion of *Xist* are used [123]. The reason why *Xist* is ectopically expressed in nuclear transfer embryos is not entirely clear. One reason could be that the *Xist* promoter is in a configuration in somatic cells that leads to activation during reprogramming. Alternatively, indirect effects could lead to *Xist* activation such as ectopic RNF12 expression. The use of siRNAs against *Xist* has further been shown to be useful for improving cloning efficiency in mice suggesting a potential application for clone production [124].

Xi reactivation has been also studied using *Xenopus* oocytes as reprogramming environment [125]. Since frogs do not possess an XCI mechanism, these cross-species experiments are harder to interpret but can be useful for addressing specific questions. Interestingly, the ability to reactivate Xi by nuclear transfer to frog eggs appears to be restricted by the developmental state of the donor nuclei. The Xi of epiblast stem cells (EpiSCs) and ES cells is reactivated upon transfer into frog eggs. In contrast, the Xi of embryonic fibroblasts or extraembryonic cells cannot be reactivated in the frog reprogramming system. Analysis of Xi chromatin composition has led to the suggestion that the histone variant MacroH2A could contribute to inhibition of reactivation in *Xenopus* oocytes. MacroH2A is enriched on the Xi of fibroblast and extraembryonic cells but is absent from the Xi in ES cells and EpiSCs. Conversely, depletion of MacroH2A in MEFs by RNA interference enhances Xi reactivation after transfer into frog eggs to some extent. This finding suggests that macroH2A contributes to the stability of the Xi but additional factors are also involved. *Xenopus* oocytes provide a biochemical system for understanding reprogramming and might be useful for understanding certain aspects of Xi reactivation. Several factors have been implicated in reprogramming including Nucleoplasmin [126], histone H1 [127], and nuclear actin [128]. Future studies will need to establish to what extent reprogramming in *Xenopus* and mouse oocytes is conserved.

### Xi reactivation by fusion with pluripotent cells

Pluripotent stem cells have been derived from preimplantation embryos or germ cell tumors. Embryonic carcinoma (EC) cells are derived from teratocarcinomas and resemble some aspects of pluripotency, as they can differentiate into cell types of the three germ layers when transplanted into immunodeficient mice. It has been observed that fusion of somatic cells with EC cells can induce reactivation of the Xi of the somatic cell [83]. Analogous experiments have also been carried out with mouse ES cells [116, 129]. These observations are a powerful demonstration of the capability of mouse ES cells that not only carry two active X chromosomes but also possess the pathways to reactivate an Xi of somatic cells. The specific chromatin environment in pluripotent cells, such as EC or ES cells, appears to permit reprogramming of epigenetic patterns of the somatic genome.

For understanding the molecular basis of this reprogramming ability, the genetic requirements for reprogramming of somatic cells by mouse ES cells have been investigated. Initial studies have shown that the de novo DNA methyltransferases *Dnmt3a* and *Dnmt3b*, *Dnmt1*, as well as the chromatin factors *Mll*, *G9a*, *Jarid2* and the nuclease *Dicer* are not required for reprogramming the somatic cell genome by ES cell fusion [130]. In contrast, the Polycomb proteins EED and SUZ12 are required for reprogramming by the ES cell fusion partner [130]. Notably, the defect in reprogramming cannot be compensated by an additional normal ES cell fusion partner, indicating that *Eed*-deficient ES cells exert a dominant effect and inhibit reprogramming [130]. It has been suggested that this inhibition could be caused by the expression of genes that are normally repressed by Polycomb activity and interfere with critical steps in reprogramming the somatic cell. It has been observed that although *Eed* is not essential for ES cell survival, it nonetheless leads to aberrant gene expression and a compromised phenotype [107]. This could suggest that reprogramming of somatic cells depends on a stable nuclear phenotype of pluripotent cells.

In addition to fusion of somatic cells with ES or EC cells, fusion with embryonic germ (EG) cells has also been shown to induce reprogramming of the somatic cell genome. EG cells can be established from PGCs and are maintained in culture as cell lines with similar morphology and differentiation potential as mouse ES cells. During PGC development, Xi reactivation is also accompanied by the erasure of genomic imprints, which makes the germ lineage a unique reprogramming system (Table 1). It has been shown that fusion of somatic cells with EG cells induces Xi reactivation and in addition leads to a loss of DNA methylation patterns associated with the control of imprinted genes [131]. In contrast, genomic imprinting is largely maintained in fusion products of somatic cells with mouse ES cells [129] or embryonal carcinoma (EC) cells [132]. These observations suggest that EG cells possess an extended reprogramming capability that has triggered interest in defining the molecular basis of these reprogramming pathways. In this regard, a recent study reporting the generation of PGC-like cells (PGCLCs) from ES cells is of considerable importance. PGCLCs can give rise to functional sperm when transplanted into the genital ridge of host embryos [133]. This in vitro system is promising to define the molecular basis of PGC development and the mechanism of epigenetic reprogramming. Taken together, a number of studies have illustrated the application of cell fusion to gain insight into epigenetic reprogramming associated with cell-type changes. However, these types of experiments also have limitations. Cell-fusion experiments produce tetraploid cells that might differ from normal diploid cell types. This concern has to be considered for the interpretation of cell-fusion experiments.

### Xi reactivation during reprogramming of induced pluripotent stem cells

Expression of the four transcription factors OCT4, KLF4, SOX2, and c-MYC can reprogram somatic cells to a pluripotent state, thereby establishing an iPS cell line [134]. It has been shown that Xi reactivation occurs at a late stage in the reprogramming process of mouse cells after the endogenous *Oct4* promoter is activated [64]. Since any cell type can be reprogrammed to an iPS cell-like state, recent studies have focused on closely related cell types for understanding the trigger for Xi reactivation. It has been shown that pre-iPS cells that are trapped in an intermediate state of reprogramming can be converted to iPS cells by using a defined medium [114]. In this system, a rapid transition to fully reprogrammed iPS cells occurs with high frequency and is accompanied by reactivation of the Xi from the pre-iPS cells to an active X chromosome in iPS cells. Similarly, the transition from mouse EpiSCs to ES cells can be accomplished by expressing *Klf4* and is accompanied by Xi reactivation [135]. Analysis of these transitions facilitates the molecular characterization of changes that correlate with Xi reactivation. Since Oct4 and Sox2 are expressed in EpiSCs [135], it is unlikely that they have a critical role in triggering Xi reprogramming. Although *Nanog* is expressed in EpiSCs, overexpression of *Nanog* leads to conversion to iPS cells and Xi reactivation [113]. In addition, REX1 expression is specific for ES cells and could therefore be a factor in either *Xist* repression or Xi reactivation [136].

It is still unclear if *Xist* repression during reprogramming is required or a correlative effect of the reprogramming process. Reprogramming in human cells and reprogramming of mouse somatic cells to EpiSCs [137] does not involve reactivation of the Xi. In both cases, pluripotent cells are generated, suggesting that induction of a pluripotent developmental potential does not require Xi reactivation. However, Xi reactivation might indicate a chromatin environment that makes reprogramming more efficient or complete. Recently, Xi reactivation in human iPS cells has been reported. When female human somatic cells are reprogrammed by expression of OCT4, KLF4, and KLF2 in culture conditions including LIF and inhibitors of GSK3 and ERK kinase activity iPS cells with two active X chromosomes can be obtained [138]. Similar results were obtained with ectopic expression of six factors (OCT4, SOX2, KLF4, c-MYC, RAR-γ, and LRH-1) in medium containing LIF and GSK3 and MEK inhibitors [139]. In addition, pre-XCI human ES cells were established from embryos in medium containing LIF in physiological oxygen atmosphere [140]. These “naive” human pluripotent cells may improve the quality of therapeutic research and regenerative medicine. Furthermore, they might provide a model for studying XCI and Xi reactivation in humans. Xi reactivation could thereby be useful for selecting human ES and iPS cells of different qualities from female donors.

Studies of genomic reprogramming in PGCs have identified additional factors that are linked to epigenetic reprogramming not only of the Xi but also of genomic imprints. *Prdm14* encodes a PR domain-containing transcriptional regulator that is highly expressed during PGCs development and has a critical role for the establishment of the germ line. *Prdm14* is required for *Sox2* upregulation during epigenetic reprogramming in PGCs [141]. Although weak and transient *Prdm14* expression is observed in blastocyst, *Prdm14* might not be required for maintaining pluripotency in ICM since Prdm14 mutant mice develop normally except for defects in the germ cell lineages [141]. Recently, Gillich et al. [142] have reported that the overexpression of *Prdm14* and *Klf2* in mouse EpiSCs can induce highly efficient conversion to ES cells and trigger Xi reactivation. These results indicate that factors identified in germ cell development might be useful for reactivating the Xi and also for enhancing the reprogramming process. Culture systems that allow manipulating the developmental state of cells such as ES cells, PGCs, and EpiSCs will be essential for understanding the molecular pathways for reprogramming epigenetic patterns in the future.

## Reactivation of genes on the Xi in development and disease

Xi reactivation is also observed in a small number of cells in the mouse extraembryonic lineages, including trophoblast giant cells [91] and parietal endoderm cells [27]. If Xi reactivation at these stages is developmentally controlled or represents stochastic events that lead to a failure of XCI maintenance is unclear at present. It further needs to be investigated if reactivation occurs chromosome-wide in these cases. In a mouse model for inducible *Tsix* expression, the number of cells that reactivate the Xi has been increased several-fold by forced repression of *Xist* [27]. Similarly, a mutation of *Eed* causes the loss of *Xist* and Xi silencing in the developing trophectoderm [143]. These observations could suggest that loss of dosage compensation can be induced and is tolerated in extraembryonic tissues of the mouse. In contrast to extraembryonic lineages, Xi reactivation is not observed in embryonic cell types. A combination of *Xist* deletion, DNA demethylation, and histone deacetylation increases the frequency of Xi reactivation in primary mouse embryonic fibroblasts [23]. Reactivation is gene-specific and likely reflects stochastic events. This has been inferred from analyzing clones of fibroblast where individual X-linked genes have been reactivated but other genes remained silenced [23]. A screen based on a targeted siRNA library has identified several candidate genes that are involved in the maintenance of XCI in somatic cells. Interference with the expression of the origin recognition complex 2 (*Orc2*) and heterochromatin protein 1 (HP1a) genes has been shown to lead to derepression of genes on the Xi [144]. *Orc2* encodes a general factor for DNA replication, thus, having additional roles in cell division. HP1a is associated with pericentric heterochromatin in the mouse and has also been observed on the Xi in some human cell lines [145]. RNA interference experiments have further implicated *macroH2A1* and *Bmi1* as factors for XCI maintenance [146]. Mutation of *macroH2A1* is compatible with normal development and female mice are healthy and fertile [147]. This finding suggests that *macroH2A1* is not essential for XCI and could indicate that other factors could compensate for mutation of *macroH2A1* in development. Similarly, a mutation of *Bmi1* is compatible with female embryo development precluding an essential role in XCI [148]. These observations suggest that a number of factors affect the stability of gene silencing on the Xi but future work will be needed to elucidate the entire chromatin configuration that underlies XCI maintenance in somatic cells.

Mouse cells with reporter genes on the Xi are a valuable tool for performing chemical screens for identifying molecules that induce Xi reactivation. These screens could be useful for identifying compounds with activity towards chromatin modifying or regulatory factors. Xi reactivation could thus provide a powerful system for studying chromatin remodeling and reprogramming. Potential applications for Xi reactivation are human diseases that are caused by gene mutations on the X chromosome. In principle, reactivation of the intact copy of the gene from the Xi in female patients could remedy the genetic defect. Ideally, this treatment would be gene-specific and targeted to the relevant cell types. However, the observation that differentiated cell types can tolerate loss of dosage compensation at least in extraembryonic tissues could also encourage chromosome-wide reactivation approaches. Reactivation of the *MeCP2* gene from the Xi has been proposed as a potential strategy for helping RETT syndrome patients [149]. RETT syndrome is a neurodevelopmental disorder that has a late onset in female patients [150]. In mice, a neurodevelopmental defect can be recapitulated by a *MeCP2* mutation [151]. Importantly, restoration of *MeCP2* function in *MeCP2*-deficient mice alleviates the neuronal phenotype [149], suggesting that restoration of *MeCP2* could also help ameliorate symptoms in RETT syndrome patients. Xi reactivation could be applied to a number of X-linked human diseases [12]. However, these approaches need to be carefully considered. Effective treatments for reactivation of the Xi are likely correlated with widespread disruption of epigenetic patterns elsewhere in the genome that could cause adverse effects. Even if side-effects are not immediate, impaired epigenetic regulation could have long-term effects and could lead to problems associated with stem cell maintenance or cell transformation. Further studies are therefore needed before such approaches can be adopted in the clinic. Considerations of limiting potential treatments to certain tissues or cell types are important. Although more work is required, Xi reactivation approaches could avoid genetic transformation with expression vectors for the defective genes and thus offer an exciting and complementary opportunity to existing strategies.

Loss of the Xi or reactivation of the Xi has also been associated with certain human tumors. In *BRCA1*-deficient breast tumors, a loss of the Xi and gain of an Xa has been observed [152, 153]. In a subset of tumors, reactivation of the Xi has been suggested as the likely cause. Elevated X:A dosage also accompanies strongly hypoploid tumor cells associated with rare human tumors ([154] and references therein). Presently, it is unclear if X-linked gene expression is selected for in certain tumors or if these observations are made as a consequence of drift of the tumor karyotype. Intriguingly, multiple X chromosomes are also reported in cases of testicular germ cell tumors [155]. These tumors appear to possess predominantly hypomethylated and active X chromosomes irrespective of *XIST* expression. The accumulation of multiple X chromosomes in these tumors has further been linked to expression of the X-linked oncogenes ARAF1 and ELK1 [155]. Taken together, these observations suggest that dosage compensation can be dynamic in certain tumors and XCI could be furthermore useful for tumor diagnosis in some instances.

## Concluding remarks and future outlook

The X chromosome undergoes repeated inactivation and reactivation during development. Reactivation of genes on the paternal X chromosome is observed in the fertilized oocyte. In the mouse, reactivation of the Xi in the developing epiblast follows imprinted inactivation in preimplantation embryo. In other mammals that do not have imprinted XCI, XCI might not be initiated before the blastocyst is formed and, hence, Xi reactivation is not required. This could also be the case in humans, but more work is needed to confirm this interpretation. During development of the female germline, Xi reactivation overlaps with a period of migration and genome-wide reprogramming in PGCs. Understanding of the mechanism of Xi reactivation could be applied for developing therapeutic strategies to cure genetic diseases that involve mutations in X-linked genes such as muscular dystrophy and RETT syndrome [149]. Cell culture models such as iPS cell reprogramming or cell fusion with ES cells provide opportunities for studying Xi reactivation. Immense progress in understanding the mechanism has been made in recent years but several questions remain to be addressed in the future.

A critical question is what the requirement for Xi reactivation is. In the mouse, dosage compensation is lost in the developing epiblast between E4.5 and E6.5 and could lead to differences in relative gene expression between male and female embryos. It will be interesting to analyze if Xi reactivation in the epiblast reflects a special epigenetic environment linked to the pluripotency of the cells. Although dosage compensation is required for female development, it appears that XCI and X:A ratios are critical only at certain developmental states. Aneuploidy involving X chromosomes is tolerated in cell culture and might even be selected for in tumors. It is not clear at the moment if the requirement for XCI involves very specific and sensitive stages in embryo development or is a general requirement for differentiated tissue cells. Accumulation of X chromosomes in certain tumors seems to be linked to oncogenic signals. These observations could provide an opportunity to understand drivers of tumor development that have, thus far, not been extensively studied. Recent analysis has identified dosage-sensitive genes on the X chromosome in mice [8]. This group of genes is precisely balanced between males and females and contains components of large protein complexes. These genes might contribute to pathways that either act at specific developmental stages or that are essential only at certain critical developmental events. Future studies of the cause and the consequences of Xi reactivation in different cell systems will advance our understanding of epigenetic regulation and genome evolution in mammals. This is important as several aspects of mammalian dosage compensation emerge to have clinical implications and could be useful for diagnosis and potential therapeutic strategies.

## Abbreviations

*Xic*: X inactivation center
XCI: X chromosome inactivation
Xi: Inactive X chromosome
Xa: Active X chromosome
PGCs: Primordial germ cells
MSCI: Meiotic sex chromosome inactivation
Xm: Maternally inherited X chromosome
Xp: Paternally inherited X chromosome
H3K27me3: Histone H3 tri-methylated on lysine 27
HDAC: Histone deacetylase
PcG: Polycomb group
H4Ac: Acetylated histone H4
5mC: 5 methyl cytosine
5hmC: 5 hydroxymethyl cytosine
ICM: Inner cell mass
ES cells: Embryonic stem cells
EC: Embryonic carcinoma cells
EpiSCs: Epiblast stem cells
iPS cells: Induced pluripotent stem cells
EG cells: Embryonic germ cells
